# Large-Range Movements of Neotropical Orchid Bees Observed via Radio Telemetry

**DOI:** 10.1371/journal.pone.0010738

**Published:** 2010-05-26

**Authors:** Martin Wikelski, Jerry Moxley, Alexander Eaton-Mordas, Margarita M. López-Uribe, Richard Holland, David Moskowitz, David W. Roubik, Roland Kays

**Affiliations:** 1 Department of Migration and Immuno-Ecology, Max Planck Institute for Ornithology, Radolfzell, Germany; 2 Department of Ecology and Evolutionary Biology, Princeton University, Princeton, New Jersey, United States of America; 3 Department of Ecology and Evolutionary Biology, University of Arizona, Tucson, Arizona, United States of America; 4 Department of Entomology, Cornell University, Ithaca, New York, United States of America; 5 EcolSciences, Inc., Rockaway, New Jersey, United States of America; 6 Smithsonian Tropical Research Institute, Balboa, Republic of Panama; 7 New York State Museum, Albany, New York, United States of America; Royal Holloway University of London, United Kingdom

## Abstract

Neotropical orchid bees (Euglossini) are often cited as classic examples of trapline-foragers with potentially extensive foraging ranges. If long-distance movements are habitual, rare plants in widely scattered locations may benefit from euglossine pollination services. Here we report the first successful use of micro radio telemetry to track the movement of an insect pollinator in a complex and forested environment. Our results indicate that individual male orchid bees (*Exaerete frontalis*) habitually use large rainforest areas (at least 42–115 ha) on a daily basis. Aerial telemetry located individuals up to 5 km away from their core areas, and bees were often stationary, for variable periods, between flights to successive localities. These data suggest a higher degree of site fidelity than what may be expected in a free living male bee, and has implications for our understanding of biological activity patterns and the evolution of forest pollinators.

## Introduction

The majority of flowering plants rely on animals to move their pollen between individuals. Among pollinating groups, bees are arguably the most important and ubiquitous [1]–[3]. The natural movement patterns of bees have proven challenging to deduce, but their correct elucidation may permit us to make testable predictions concerning mutualisms, bee biology and parasitism, and the remarkably rapid radiation of angiosperms. Additionally, pollination is a key component to insect ecosystem services. Given the escalating rate of human interference, and the potential for deterioration of pollination services [4], [5], it is critical that we start to understand the complexities of these relationships.

Bee flight range has been estimated primarily from feeder training and homing experiments or mass marking [5], [6], [7], [8], it is not known how well these reflect natural movements of bees, especially in tropical forests [but see 9], [10]. In addition, bee mobility and ecology, as they locate resources and avoid stress sources, has never been adequately documented in a natural environment. Until recently [11], the only accurate and repeated studies of bee foraging activities involved social or communal bees, which demonstrate a bell-shaped space-use curve, centred on their nest [12], [13].

Ecological services provided by bees, while not currently under debate, are often without direct evidence. This is remarkable and constitutes a substantial scientific gap, yet for bees in general, long-distance tracking methodologies have never been available. The primary method to date has involved marking bees and re-sighting them at baits spread throughout a given study area, or as a complementary strategy, by demonstrating that pollen has been dispersed a certain distance by a presumed pollinator. This has revealed surprisingly long movements of up to 1.8km in Gabon forest for *Apis mellifera* and 2.1km in Panama for *Melipona* and *Cephalotrigona* [14], [15]. However, this method relies on attractive baits or known bee nests, and is limited by the distribution of baiting stations. It is thus difficult to accurately reconstruct the intricacies of foraging and flight movement patterns using these methods. The best active-tracking system for insects so far—harmonic radar [16], only allows researchers to follow individual bees in open habitat where radar beams are not blocked by vegetation [17], and where bees do not routinely fly outside the reference areas.

Radio telemetry has been successfully applied to study the movement of individual insects such as carpenter bees [11], beetles [18], Mormon crickets [19] and migrating dragonflies [20], [21]. Here we make use of miniaturized radio transmitters to study the foraging ranges of individual orchid bees without the bias of baiting stations.

Euglossine ‘orchid’ bees are restricted to the tropical Western Hemisphere. Their 200 species influence the evolution and maintenance of not only orchids but diverse tropical forest trees, vines, shrubs and herbs [22]–[25]. Male euglossine bees are attracted to, and collect, fragrant chemicals produced by rotting organic material, flowers, and other plant parts [26], [27]. To collect the fragrances, food, or nesting material, the bees may travel extraordinarily large distances (for their body size) and contribute significantly to plant long-distance pollination, even across a fragmented (open patches interspersed with forest) landscape [28]. Long-distance movement of bees may be significantly impacted by how much heat bees tolerate [29]. Thus it is of interest that Janzen [9] observed pollen-loaded *Eulaema* females to cross the open water of the Panama Canal. That represents a large distance, but more importantly, extensive exposure in the heat of the day. When Janzen [9] translocated six female *Eufriesea surinamensis* 20–23km from their nests in lowland Costa Rican rain forest, 4 returned on the same day, one bee within 65 minutes. Dressler [23] noted that pollinaria of male *Eulaema* bees caught on Barro Colorado Island, Panama, likely originated from forest 40 km away.

Euglossine bees may also ‘trapline’ flowers over large distances in the rainforest [9], [26]. However, interpretations of traplining (when bees visit flowers in a stable, repeatable sequence) are often based on circumstantial evidence, basically taken at stopping points within an unknown activity circuit, like the observations made by Janzen [9] who initially observed 3 individual females to return for 3 consecutive days to the site of an experimentally removed flower, and one individual *Eulaema* female that returned for 5 days at around 7 a.m. to a *Heliconia* plant. However, observing a bee making repeated appearances at a single flower demonstrate only that it has spatial memory for the location, whereas traplining suggests that it visits a series of sites in a stable, repeatable sequence. Hence, observing repeated visits by an individual bee to a flower at a particular location is not sufficient to demonstrate traplining in a definitive manner.

**Figure 1:**
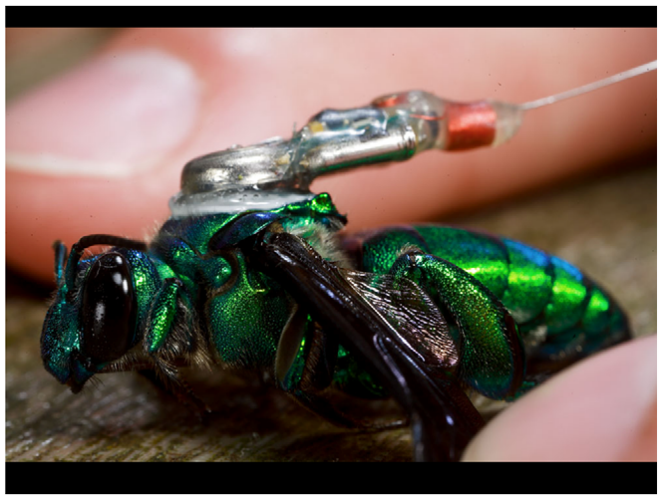
Attachment of a micro-radio transmitter to a male orchid bee *Exaerete frontalis*. Transmitters (300mg) were attached to the bee's thorax by the battery, using eyelash adhesive inside, and minute amounts of superglue around the outside of the battery. The angle at which the transmitter stuck off the bee was somewhat variable and usually lower than in this picture, i.e., the transmitter was closer to the bee's abdomen. Picture by Christian Ziegler.

The aim of our study was to extend these observations to understand spatial distribution of euglossines, irrespective of chemical baiting stations or fragrance collection by males. We selected the males of a cleptoparasitic euglossine species, *Exaerete frontalis* because of their abundance, large size (mean of 11 males without nectar meals = 612.4 mg, range 493.5–694.5), and their ability to carry the still relatively heavy radio transmitter load (300mg, Fig. 1) [30], [31]. Unlike most euglossines, *Exaerete* females lay their eggs in nests of other euglossine bees. Male *Exaerete*, like euglossine males in general, do not occupy a nest during adulthood, but they share a nocturnal resting site, and diurnal perching or display sites for courtship, with other males during some portion of their life; they feed on nectar-producing flowers and most visit orchid flowers for fragrances [25], [32], [33].

## Results

### Success of radio tracking individual bees

Of the 16 radio tagged bees, we lost 4 individuals during the same day and never found them again. The other individuals were observed for 5±1.9 (SD) days and we gathered 8±5 (range 1–19) independent location estimates per individual. In addition to ground tracking, we collected 14 location estimates of 10 individuals during two helicopter tracking overflights. A total of 5 bees lost their transmitters after some time, usually a few days, and we found the transmitter but no bee. We also found 4 dead bees, three on the ground and one in a spider hole in the ground. Five bees retained their transmitter and were confirmed alive at the end of the study (after 10 days of transmitter life). During our study, three of the tracked bees were observed over a period of 3–4 days, returning to the same trees in their home/foraging range, at similar times. Unfortunately, we obtained very few visual observations, either when they were flying or when they repeatedly returned to favoured trees.

### Home ranges and movement of individual bees

**Figure 2:**
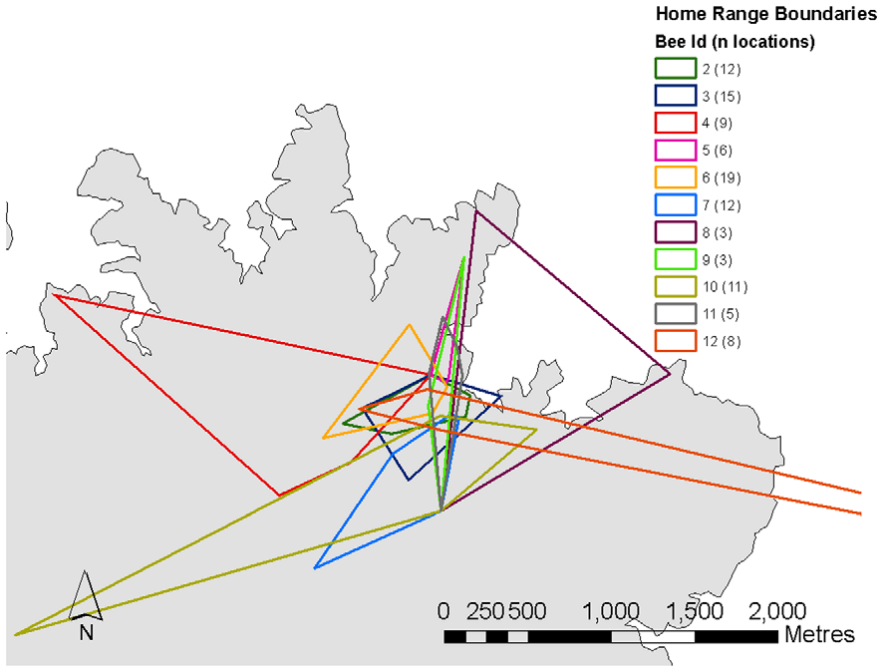
Home ranges of 11 orchid bees on Barro Colorado Island. While some home ranges are small and centred around the capture sites situated in the NE of the island. Others use very large ranges, with one even including a site off the island on the other side of the Panama canal. These data are available at www.movebank.org.

**Figure 3:**
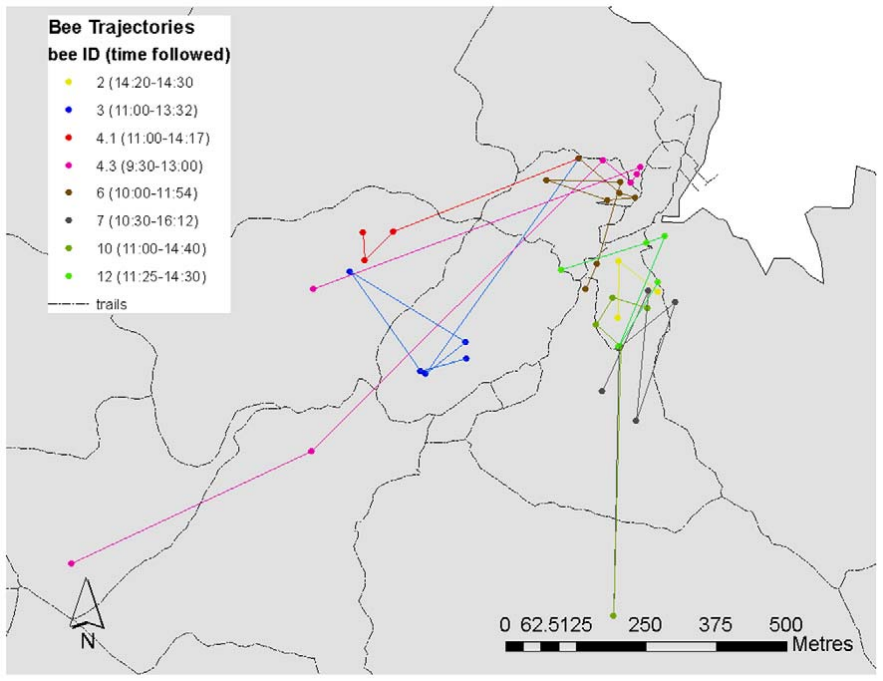
Flight trajectories of 7 orchid bees. The different colors indicate different flights of orchid bees, with lines connecting consecutive locations (dots). Bees were stationary for variable lengths of time and then flew to a new site where again they remained stationary.

The minimum home range size of 11 individual bees (for one of 12 bees we only had one location fix), determined by the minimum convex polygon method, was on average 45±40 (SD) ha and ranged from 4 to 700 ha (Fig. 2). This is probably an underestimate because home range increased with the number of locations and it levelled off for most bees between 10–15 locations. Only five of our animals had >10 locations, and their home range size was 35.8±29 ha. We expect that our detailed observations were biased towards individuals with slightly smaller home ranges. We followed 8 individual flights of 7 bees for a straight-line distance of 846±195 m, which amounted to a total linear distance travelled (including all linear travel segments) of 1516±340 metres (Fig. 3). We were in radio contact with the bees during the entire flight and they indicate accurate estimates of movement speeds. The bees moved at an average speed of 9.5±1.7 m/min (including intermediate resting times). Their flights lasted 192±37 min (3h 12 min). The two longest tracking results were 1.9km (5.1hrs) and 1.2km (5.7hrs). The average turning angle of individual movement trajectories was 102±9.5 degrees. All tracking locations from this study are available at www.movebank.org.

## Discussion

Radio telemetry of large orchid bees confirmed previous suggestions that males have large home ranges [9], [34], [35]. We also found evidence that orchid bees may trapline floral resources, as previously thought, because individual bees visited specific sites on successive days. This is far from definitive evidence for traplining, but it shows the potential for radio-telemetry as a methodology to collect the data necessary to discriminate among alternative explanations. Such large flight distances may be typical for large bees, like the carpenter bees tracked, in Africa, with similar transmitters [11].

Due to the manipulative nature of our study, i.e., attaching relatively heavy devices to small but powerful flying insects, the outcome must be interpreted carefully. Previous insect radio telemetry studies were conducted either with crawling insects such as Mormon crickets [19], or with very large insects that have strong flight capacities, such as certain large beetles [18] or dragonflies [20]. By extending radio telemetry to bees that are scarcely a few times heavier than transmitters we obtained credible results because orchid bees are known for their flight capabilities [30], [31], [36]. For example, Dudley [30] demonstrated three species of orchid bees hovering in heliox (a mixture of helium and oxygen) dramatically increased wing power output, compared to flight in normal air. Similarly, Darveau et al. [31] showed orchid bees maintain extraordinarily high flight efficiency and convert nectar into flight energy at impressive rates. Thus we presume that orchid bees carrying a transmitter can increase their lift and mass-specific power output, although associated tradeoffs in speed and efficiency might occur [37]. Nevertheless, we will not know – until better observational methods and smaller transmitters become available – whether the current results represent natural behaviour for *Exaerete* or are minimal movements, restricted by the weight of the transmitter. Alternatively, the observed ranges could have been confined by the 16km^2^ of available island habitat, although the flight of one of our bees across the lake and back to the island suggests this is not the case. That bee was first tagged near the middle of BCI on 4 March and was tracked that day in that general area. The following day it was located using aerial tracking ∼5km away across the Panama Canal, in the forests of Soberania Park. The minimum over-water distance between BCI and Soberania in this direction is 1400m. We could not follow this bee's movements in Soberania in detail, but found it back again in the centre of BCI on 9 March.

The distances travelled by the bees within their home ranges are approximately consistent with expectations of foraging ranges based on various field tests for smaller bees [5]. Using the body sizes from *E. frontalis* specimens and fitting it to Greenleaf et al.'s formula [5], we predicted ca. 9–10 km foraging range, equivalent to that of an orchid bee that flew across the Panama Canal. However, such a comparison is only valid if one assumes that feeder training and/or homing experiments represent a routine distance travelled by individual bees. In our observations several orchid bees appeared to regularly travel large distances (many kilometres). It is unclear how well such extrapolations can match the normal flight and foraging behaviour of bees. It is also unclear, as stated above, whether the small size of Barro Colorado Island influences movement distances, although smaller forest fragments are increasingly common in many parts of the world. Future investigations of basic movement patterns are needed to see if the home range sizes we observed for orchid bees on BCI are typical of bees of this size. While documenting bee movement across inhospitable areas, such as the Panama Canal, our data would add strong support to the claim that bees are indeed major agents of gene flow, connecting plants over fragmented landscapes [28], [38]–[40].

The range of home range sizes recorded for our 12 *E. frontalis* males is compatible with our hypothesis that these males could be a mix of resident (reproductive perch owner) animals using small areas, combined with transient individuals that cover more ground [9]. Alternatively, we propose two other, non-exclusive hypotheses: firstly, all individuals could use multiple core areas widely separated by unused (or little used) areas. Limitations in our current methodology, particularly the comparatively short battery life, mean we may only have detected one core area in any detail. Secondly, orchid bees use only one core area extensively but have an extremely large periphery which they visit to gather rare resources, such as floral fragrances. These three patterns of individual movement have different implications for pollination services, and each demand testing with more bee tracking, provided radio-transmitters continue shrinking in size and mass.

Whichever scenario turns out to be true in future investigations, the data confirm that male orchid bees habitually travel a distance that can help connect widely-dispersed orchids or other plants which they alone pollinate, and that produce a few short-lived flowers daily, over an extended time [25]. Thus our data support the hypothesis that orchid bees are key evolutionary players in allowing orchids and other tropical plants evolve into diverse taxa that are each spatially rare and thus require long-distance pollination [41]–[45].

Our data also demonstrate that it is now feasible to study movement patterns *within* home range in a direct manner, as never before, among individual bees as small as 0.6 grams. We suggest this technique will yield data currently unappreciated in agriculture [46], conservation [47], economy [48] and general biology [18], [19], [49].

## Materials and Methods

We captured 17 male orchid bees (*E. frontalis*) using insect nets and chemical baits between Mar-3 and Mar-10, 2007 at six sites on Barro Colorado Island (BCI), Republic of Panama (79°50′W to 9°09′N latitude). To attract male *E. frontalis*, small pieces of paper were soaked with 1,8-cineole or methyl salicylate (source: Merck & Co. Inc., NJ, USA), fundamental compounds in orchid floral fragrances [23], [50], [51], and attached to tree trunks at ca. 1.2 metres height. We did not observe any adverse responses to the glue as recently reported for corn rootworms (*Diabrotica sp.*) [52]. All individuals were relatively young adults, as judged by the general lack of wing damage. Collected individuals were fitted with small (300mg) radio transmitters (Sparrow Systems, Fisher, IL, 2 radio pulses per second, 378 MHz, antenna length 42mm) at the dorsal thorax using minute amounts of a combination of eyelash adhesive (Andrea glue, American International Industries, Commerce, CA) and superglue (Krazy Glue, Elmers, OH). One person held the bee at the thorax while another person attached the transmitter (Fig. 1). Bees studied here were not weighed to minimize handling and stress before transmitter deployment. To estimate body size in *E. frontalis*, we used ten randomly selected pinned males from Panama, and measured their minimum inter-tegular distance to the nearest 0.05 mm (mean+−SD = 5.56+−0.289 mm).

We initially attempted to cool bees for handling purposes, but stopped doing this during transmitter attachment because we found that cooled bees needed a long time period (hours) to recover full flight capacity. During tagging, bees had to be held tight between two fingers such that ‘buzzing’ was reduced to a minimum. If bees buzzed while the glue was still hardening, the transmitter invariably moved and/or fell off and had to be reattached/realigned. Once the glue had set, buzzing did not affect the transmitter attachment any longer. Tagged bees took off within 3 minutes of release, and initially often flew away with some drop in altitude (see supplementary Video S1). However, we subsequently observed the same individuals fly through the forest and visually could not detect a difference between the flight of tagged bees versus natural flight. We were unable to quantify how the transmitter load affected flight performance. Because orchid bees are strong flyers [30], [31], [36], their ability to carry a 300 mg radio-transmitter with little apparent difficulty is not too surprising.

**Figure 4:**
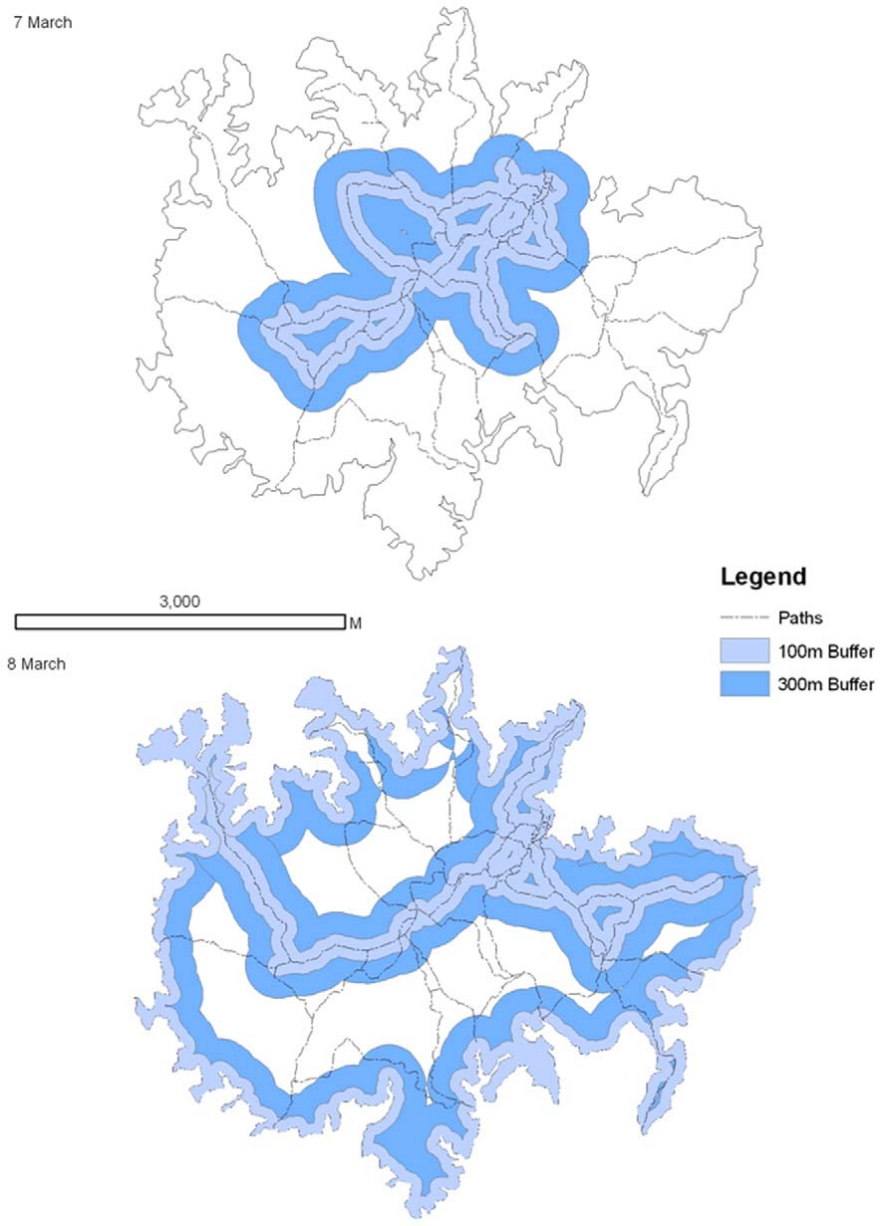
Radio tracking sampling effort on Barro Colorado Island. The outline of the island is shown, as well as the total area in which a micro-transmitter would have been detected. Under worst-case conditions (e.g., bee in a tree hole), a transmitter can only be observed for 100 metres. Usually, detection range is ca. 300 metres even in dense forest understory.

During tracking sessions bees were tracked continuously by two tracking teams from the ground using conventional radio telemetry techniques. When possible, we also located animals using two aerial surveys conducted from a helicopter platform equipped with an external receiver antenna. Whenever the ground team received a signal from a transmitter, their own location (via GPS or trail markers) and the signal's compass direction were noted. Usually in less than ca. 5 minutes (only rarely between 5 to 20 min), a second compass direction was determined from another vantage point to allow us to estimate the position of the bee via cross-directional techniques (only a total of 8 fixes were by biangulation, for the vast majority of fixes we used at least triangulation, thus providing low error on location estimations). We call these data location observations, and consider the bee in a new location whenever the individual moved for more than ca. 100 metres. On several occasions we got visual contact with individual tagged bees, but observations were too scattered to reveal quantitative behavioural information. We stopped surveying individual movements after 10 days of transmitter attachment because transmitter batteries were expected to run out at approximately that time. During ground tracking exercises, we covered the entire island of BCI via trails or from a boat going around the shores of the island (Fig. 4). Nevertheless we may have failed to detect bees even if they were present. We conducted one complete terrestrial tracking survey every day. In addition to locating bees we followed the trajectories of 7 individual bees on 8 occasions (Fig. 3) for up to 342 minutes. During these individual observations the signal of the bees was always in range of the observer.

In all analyses, we report population means±SE except when noted (SD = standard deviation). We used SPSS for Windows 12.0 for all statistical calculations and BIOTAS 2.0 (www.ecostats.com, Ecological Software Solutions Inc.) for range analyses. To determine the home/foraging range size, we used the minimum convex polygon method [53]. Because we do not know the details of the bees spatial behaviour, we call the bee's individual movement ranges ‘home range’ for convenience.

## Supporting Information

Video S1 Slow motion footage of a radio-tagged bee (Bee ID 7) flying away in its release. Note that the animal initially drops altitude in initial take-off, but recovers to fly quickly and successfully through the complex forest. Please download and un-zip to view in Quicktime (.mp4 format).
(9.34 MB ZIP)
